# Association of platelet to high-density lipoprotein cholesterol ratio with hyperuricemia

**DOI:** 10.1038/s41598-024-66747-x

**Published:** 2024-07-08

**Authors:** Laisha Yan, Xiaoyan Hu, Shanshan Wu, Shunying Zhao

**Affiliations:** Department of Cardio Surgery Intensive Care Unit, Ningbo Medical Centre Li Huili Hospital, Ningbo, China

**Keywords:** Platelet, Cholesterol, Uric acid, NHANES, Endocrinology, Nephrology

## Abstract

The platelet/high-density lipoprotein ratio (PHR) has been identified as a significant indicator of inflammation and a hypercoagulable state, demonstrating a strong link with the severity of nonalcoholic fatty liver disease (NAFLD) and metabolic syndrome (MetS). However, its correlation with hyperuricemia has not yet been documented. This study utilized a cross-sectional design, analyzing data collected from the National Health and Nutrition Examination Survey (NHANES) between 2007 and 2016 in the United States. The platelet/high-density lipoprotein ratio (PHR) was determined by dividing the number of platelets (PLT) by the level of high-density lipoprotein cholesterol (HDL-C). We employed multivariable logistic regression analyses, generalized additive models, and subgroup analyses to investigate the correlation between PHR and hyperuricemia. The study revealed a hyperuricemia prevalence of 18.56%. Analysis indicated a significant positive correlation between PHR and the risk of hyperuricemia (OR 1.11, 95% CI 1.08, 1.14). This correlation remained consistent across different subgroups including age, ethnicity, gender, and body mass index (BMI). Smooth curve fitting demonstrated a saturation effect between PHR and the risk of hyperuricemia. PHR is positively correlated with hyperuricemia and may serve as a novel biomarker for predicting the onset of this condition. Additionally, targeted interventions to improve PHR might help reduce the incidence of hyperuricemia.

## Introduction

Serum uric acid (SUA) represents the final product of purine breakdown in humans. Initially, purine is metabolized to hypoxanthine, which is then oxidized by xanthine dehydrogenase (XDH) to xanthine before further oxidation to uric acid (UA)^1^. Elevated levels of SUA can induce endothelial dysfunction, affecting vascular tone, thrombosis, inflammation, and oxidative stress^2^. High SUA levels can trigger gout and play a significant role in the development of cardiovascular diseases, including hypertension, atrial fibrillation, heart failure, and coronary artery disease. They are also closely associated with diabetes and metabolic syndrome (MetS)^3–8^. Despite a lack of extensive clinical study evidence, UA-lowering treatments have shown clinical significance in managing chronic kidney disease, blood sugar control, and cardiovascular outcomes^9–11^. In the United States, during 2015–2016, 47.1 million adults met the criteria for hyperuricemia, with a prevalence rate of 20.1%^12^. This condition and its associated comorbidities significantly increase the public health burden, highlighting the importance of early prediction, identification, and treatment of hyperuricemia.

Hyperuricemia is closely associated with coagulation abnormalities. SUA, through reactive oxygen species (ROS)-mediated oxidative stress, induces an inflammatory state and vascular damage, triggering thrombosis^13^. Platelets (PLT) serve a crucial function in the coagulation process, and several studies have demonstrated an independent correlation between PLT counts and the width of the PLT distribution representing the activation of PLT and with SUA^14–17^. The lipid profile associated with hyperuricemia typically features reduced concentrations of high-density lipoprotein cholesterol (HDL-C) and elevated levels of triglycerides (TG), total cholesterol (TC), and low-density lipoprotein cholesterol (LDL-C), where HDL-C is regarded as a protective factor against elevated SUA levels^18^. Given these factors, a close relationship between the platelet/HDL-C ratio (PHR) and hyperuricemia is hypothesized. As a marker of inflammation and a hypercoagulable state, PHR has been found to be closely related to the severity of nonalcoholic fatty liver disease (NAFLD) and MetS, yet its correlation with hyperuricemia remains unexplored^19–22^.

Thus, this research employs data from the National Health and Nutrition Examination Survey (NHANES) to explore the connection between PHR and hyperuricemia.

## Methods

### Study population

The NHANES is a project aimed at evaluating the health and nutritional condition of the American population. NHANES combines interviews with physical examinations to collect data on health, socioeconomic status, and diet, among other areas. This data helps identify disease prevalence, risk factors, and nutritional status, providing crucial information for public health policies and health measurement standards such as height and weight.

**Figure 1:**
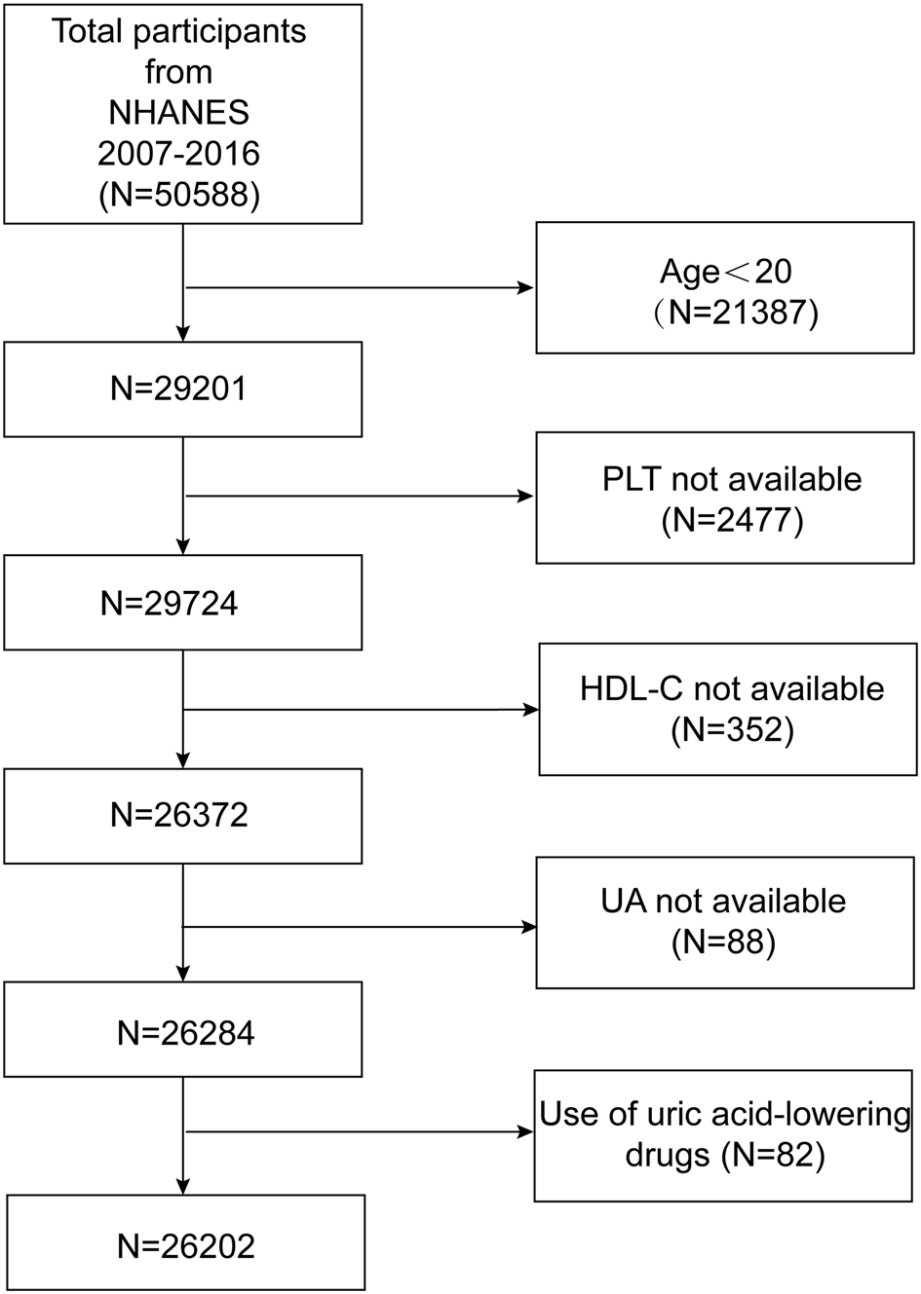
Flowchart of participant selection. *HDL-C* high-density lipoprotein cholesterol, *SUA* serum uric acid, *PLT* platelets, *NHANES* National Health and Nutrition Examination Survey.

This study analyzed data from the 2007–2016 cycle with 50,588 participants. After excluding participants under the age of 20, those using uric acid-lowering medications, or lacking data on HDL-C, PLT, or UA, a total of 26,202 subjects remained eligible for analysis. Figure 1 outlines the criteria for inclusion and exclusion used in the study.

### Study variables

The exposure variable, PHR, is calculated as the ratio of PLT (1000 cells/uL) to HDL-C(mg/dL). Trained researchers collect blood samples from each participant to perform a complete blood cell count using VCS (Volume, Conductivity, and Scatter) technology. From 2007 to 2012, the hematology analyzer used was the Beckman Coulter MAXM; however, from 2013 to 2016, the Beckman Coulter DXH 800 was employed for this purpose. Serum samples were stored at minus 30 degrees Celsius for the direct measurement of HDL-C using immunoassay methods. During the 2007–2012 cycle, HDL-C testing was conducted with the Roche Modular P Chemistry Analyzer, while in the 2013–2016 cycle, both the Roche Modular P and the Roche Cobas 6000 chemistry analyzers were utilized.

Hyperuricemia, the outcome variable, is defined as a SUA level exceeding 420 μmol/L in adult males and 360 μmol/L in adult females.

Traditional risk factors for hyperuricemia were considered as potential confounders and included in the final multivariate logistic regression model. Covariates encompassed demographic, health, and biochemical indicators: gender, age, race, education level, Body Mass Index (BMI), hypertension, diabetes, hyperlipidemia presence, alcohol consumption, smoking status, and levels of creatinine (Cr), alanine aminotransferase (ALT), aspartate aminotransferase (AST), Hemoglobin A1c (HbA1c), estimated glomerular filtration rate (eGFR), LDL-C, and hemoglobin (HGB). Hypertension, diabetes and hyperlipidemia were self-reported through questionnaires. Smoking was characterized by the consumption of more than 100 cigarettes in a person's lifetime, while alcohol intake was defined as consuming over 12 alcoholic beverages annually. The eGFR was calculated utilizing the CKD-EPI formula^23^.

### Statistical analysis

Following the guidelines of the Centers for Disease Control and Prevention, sample weights were incorporated to address the complex probability sampling of NHANES, ensuring adequate national representation, particularly in terms of oversampling in certain survey cycles.

Continuous variables are presented as mean ± standard deviation (SD), while categorical variables are depicted as numbers (percentages). To evaluate differences among participants divided by PHR tertiles, a weighted linear regression model and chi-square tests were employed. To explore the link between PHR and hyperuricemia, multivariate logistic regression analyses were conducted across three distinct models: Model 1, which did not include any adjustments; Model 2, which adjusted for factors such as gender, age, and race; and Model 3, which incorporated adjustments for all the covariates mentioned earlier. A sensitivity analysis was also carried out, in which PHR was treated as a categorical variable, and the median value of each category was used as a continuous variable to evaluate linear trends. Subgroup analyses were conducted to assess the robustness of the outcomes and examine interactions, ensuring a thorough investigation of the data. Potential nonlinear relationships were investigated using generalized additive models and smoothing curves, with two-phase linear regression applied to pinpoint significant trend changes at inflection points. The threshold for statistical significance was established at *P* < 0.05. All analyses and visualizations were conducted using R version 4.2.0 and Empower Stats version 4.0.

### Ethics statement

The research involving human participants underwent a thorough review and received approval from the Research Ethics Review Board of the NCHS. All patients or participants gave their written informed consent to be part of this study.

## Results

### Subject characteristics

Table 1 displays that a total of 26,202 participants were included in the study. The mean age of the cohort was 47.36 ± 16.85 years, with 48.21% males and 51.79% females. The ethnic composition was as follows: 8.64% Mexican American, 5.71% Other Hispanic, 67.31% Non-Hispanic White, 10.69% Non-Hispanic Black, and 7.65% Other races. The average PHR level was 4.99 ± 2.07. There were 4,861cases identified with hyperuricemia, representing 18.56% of the total population. Individuals with higher PHR levels tended to be younger males, more likely to smoke, less likely to drink alcohol, and had lower levels of education. They also had a higher likelihood of being obese, diabetic, and suffering from hyperlipidemia and hypertension. Additionally, these individuals exhibited lower AST levels, and higher levels of HbA1c, LDL-C, SUA, GFR and ALT.

**Table 1:** Baseline characteristics of study participants.

Characteristics	PHR tertiles			*P* value
	Tertiles 1	Tertile 2	Tertile 3	
PHR range	0.239–3.958	3.958–5.524	5.524–41.191	
N	8730	8735	8737	
Age, years	50.27 ± 17.66	46.95 ± 16.82	44.72 ± 15.48	< 0.0001
Gender, %				< 0.0001
Men	39.80	50.76	54.41	
Women	60.20	49.24	45.59	
Race				< 0.0001
Mexican American, %	6.05	8.77	11.21	
Other Hispanic, %	4.29	5.96	6.96	
Non-Hispanic White, %	70.25	67.58	63.95	
Non-Hispanic Black, %	11.84	10.44	9.75	
Other races, %	7.56	7.26	8.13	
Education level				< 0.0001
Less than 9th grade, %	5.14	5.78	6.65	
9–11th grade, %	9.21	10.68	13.34	
High school graduate, %	19.26	22.41	24.38	
Some college or Associate of Arts degree, %	29.95	31.64	33.18	
College graduate or above, %	36.44	29.49	22.45	
BMI, kg/m^2^	26.59 ± 5.89	28.90 ± 6.48	31.35 ± 7.09	< 0.0001
LDL-C, mmol/L	2.85 ± 0.87	3.03 ± 0.92	3.03 ± 0.92	< 0.0001
Cr, mmol/L	77.56 ± 35.94	78.21 ± 27.70	78.24 ± 32.26	0.2873
AST, U/L	26.38 ± 16.20	25.05 ± 15.09	25.92 ± 16.11	< 0.0001
ALT, U/L	23.69 ± 20.79	24.81 ± 17.14	28.32 ± 21.07	< 0.0001
HbA1c, %	5.50 ± 0.75	5.61 ± 0.91	5.79 ± 1.07	< 0.0001
GFR, mL/min/1.73 m^2^	91.18 ± 22.41	93.82 ± 21.72	96.23 ± 21.77	< 0.0001
Diabetes, %	7.01	9.19	12.13	< 0.0001
Hypertension, %	29.87	30.96	34.52	< 0.0001
Hyperlipidemia, %	33.56	36.34	39.91	< 0.0001
Drinkers, %	78.25	77.56	75.77	0.0004
Smokers, %	41.94	43.73	48.14	< 0.0001
SUA, umol/L	303.82 ± 80.87	323.72 ± 82.27	341.22 ± 84.18	< 0.0001

*BMI* body mass index, *SUA* serum uric acid, *Cr* creatinine, *AST* aspartate aminotransferase, *ALT* alanine aminotransferase, *eGFR* estimated glomerular filtration rate, *HbA1c* hemoglobin A1c, *LDL-C* low-density lipoprotein cholesterol.

### Association between PHR and risk of hyperuricemia

Table 2 presents the results of multivariable logistic regression analyses. In the unadjusted model, PHR was positively correlated with the risk of hyperuricemia (OR 1.12, 95% CI 1.10, 1.13). This positive correlation persisted in both the minimally adjusted model (OR 1.16, 95% CI 1.14, 1.18) and the fully adjusted model (OR 1.11, 95% CI 1.08, 1.14), even after adjusting for confounders. When PHR was divided into tertiles, compared to the reference group, the odds ratios for hyperuricemia for participants in the second and third tertiles were 1.29 (95% CI 1.12, 1.49) and 1.78 (95% CI 1.54, 2.06), respectively, with a significant trend observed across the tertiles of PHR (trend *P* < 0.05).

**Table 2:** Association between PHR and the risk of hyperuricemia.

PHR	Hyperuricemia OR (95% CI), *P* value		
	Model 1	Model 2	Model 3
Continuous PHR	1.12 (1.10, 1.13), < 0.0001	1.16 (1.14, 1.18), < 0.0001	1.11 (1.08, 1.14), < 0.0001
Tertile 1	Reference	Reference	Reference
Tertile 2	1.30 (1.20, 1.41), < 0.0001	1.46 (1.34, 1.58), < 0.0001	1.29 (1.12, 1.49) 0.0003
Tertile 3	1.87 (1.73, 2.02), < 0.0001	2.30 (2.12, 2.50), < 0.0001	1.78 (1.54, 2.06), < 0.0001
*P* for trend	< 0.0001	< 0.0001	< 0.0001

The analytical models were defined as follows Model 1: unadjusted; Model 2: adjusted for sex, age, and race; and Model 3: sex, age, race, education, BMI, hypertension, diabetes mellitus, hyperlipidemia, smoking status, drinking status, Cr, eGFR, AST, ALT, HbA1c, LDL-C.

**Figure 2:**
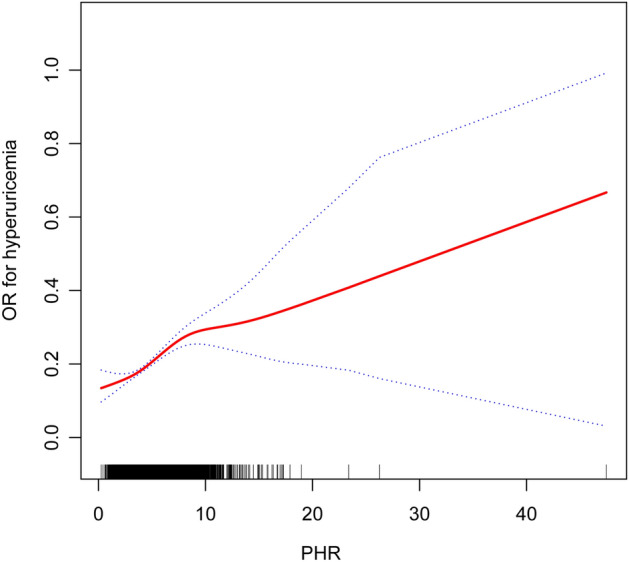
Subgroup analysis of the correlation between PHR and the risk of hyperuricemia. In addition to stratification variables, each subgroup analysis was adjusted for sex, age, race, education, BMI, hypertension, diabetes mellitus, hyperlipidemia, smoking status, drinking status, Cr, eGFR, AST, ALT, HbA1c, LDL-C.

Subgroup analyses demonstrated a consistent positive correlation between PHR and the risk of hyperuricemia across stratified participant groups based on gender, age, ethnicity, BMI categories (< 25 kg/m^2^, 25–30 kg/m^2^, ≥ 30 kg/m^2^), smoking status, alcohol consumption, hypertension, diabetes, and hyperlipidemia (Fig. 2).

**Figure 3:**
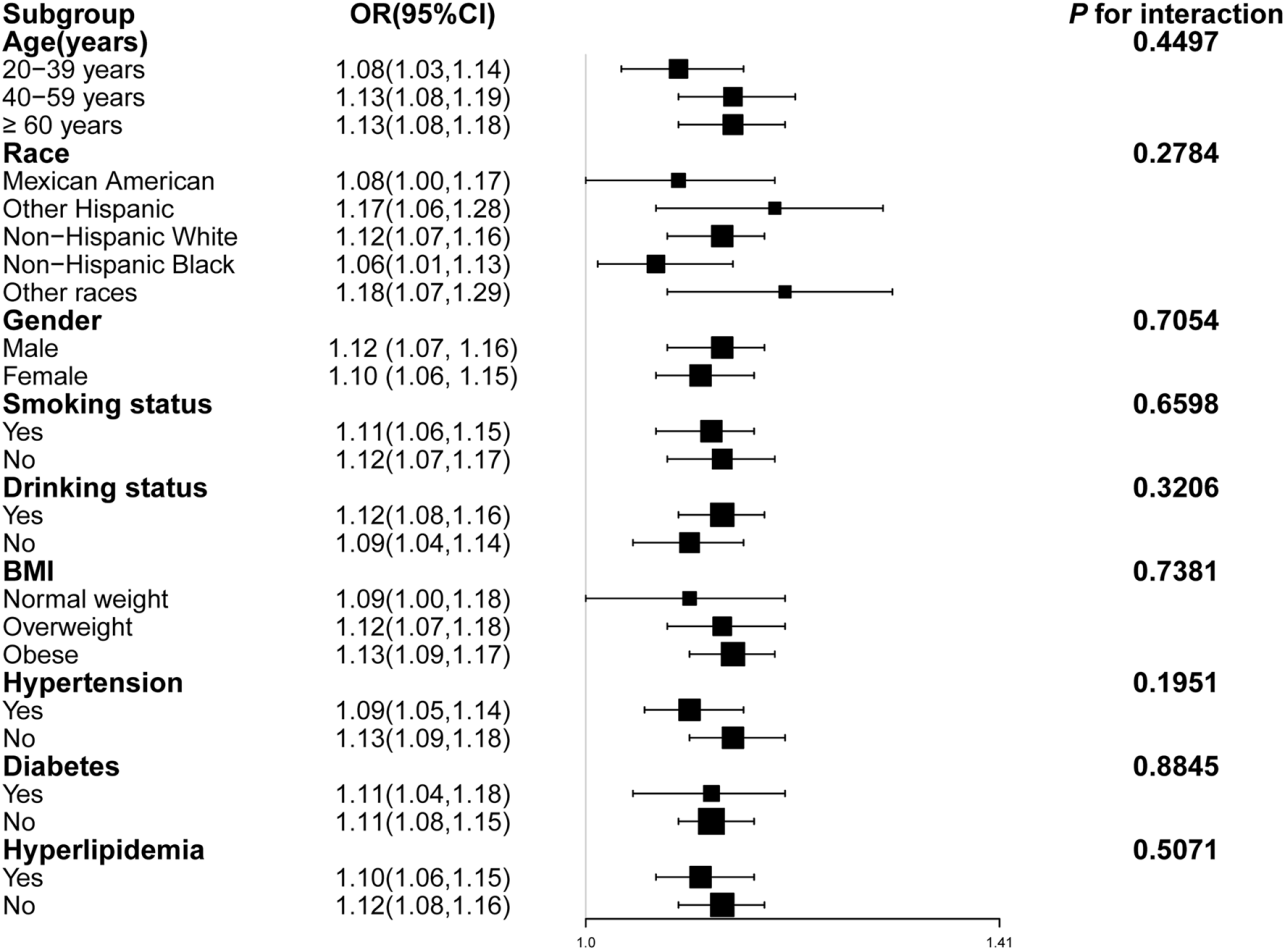
The nonlinear relationship between the PHR and the risk of hyperuricemia. All models were adjusted for sex, age, race, education, BMI, hypertension, diabetes mellitus, hyperlipidemia, smoking status, drinking status, Cr, eGFR, AST, ALT, HbA1c, LDL-C.

Figure 3 illustrates the nonlinear relationship between PHR and the prevalence of hyperuricemia via a smoothed curve fit using a generalized additive model. An inflection point was identified at 8.18 using a two-stage linear regression model (Table 3). Below this inflection point, a lower PHR corresponds to a reduced risk of hyperuricemia.

**Table 3:** Threshold analysis of the effect of PHR on hyperuricemia using two-piece linear regression models.

Hyperuricemia	OR (95%) *P* value
PHR	
Inflection point	8.18
< 8.18	1.15 (1.11, 1.19) < 0.0001
> 8.18	1.02 (0.95, 1.08) 0.6285
Log likelihood ratio	0.006

All models were adjusted for sex, age, race, education, BMI, hypertension, diabetes mellitus, hyperlipidemia, smoking status, drinking status, Cr, eGFR, AST, ALT, HbA1c, LDL-C.

## Discussion

This study is the first to assess the relationship between PHR and hyperuricemia. Results show a positive correlation between PHR and the risk of hyperuricemia, which remains significant even after adjusting for a variety of confounding factors such as ethnicity, age, educational level, and BMI. Subgroup analysis and interaction tests indicate that this positive correlation is stable across different demographic environments. Smooth curve fitting reveals a saturation effect between PHR and the risk of hyperuricemia, suggesting that an appropriate range of PHR (less than 8.18) may be beneficial for assessing the risk of hyperuricemia.

A previous study investigated the relationship between the PHR and MetS, revealing that PHR correlates with all five characteristics of MetS: abdominal obesity, hypertriglyceridemia, low HDL-C, hyperglycemia, and hypertension. Moreover, the level of PHR increases with the severity of MetS^19^. There is a significant bidirectional association between hyperuricemia and MetS. A four-year longitudinal study demonstrated that hyperuricemia is a significant and independent predictor of MetS in subjects, with the risk of MetS escalating alongside the increase in SUA levels^24^. On the other hand, MetS and its five components are significantly associated with the incidence of new-onset hyperuricemia, with the incidence rate rising with a rise in the count of MetS components^25^. This mechanism might be due to microvascular damage associated with the elevated blood pressure in MetS enhancing UA production, while the hyperinsulinemia related to MetS increases the reabsorption of UA by the proximal renal tubules^26^. Since MetS and hyperuricemia share common features like inflammation, insulin resistance, and a hypercoagulable state, it is therefore logical to propose that PHR is closely related to hyperuricemia.

The relationship between PHR and hyperuricemia involves complex pathophysiologic mechanisms that are not yet fully understood. First, there is a negative correlation between HDL-C levels and renal function^27^; lower HDL-C levels indicate a decrease in eGFR, which may lead to decreased UA excretion.

Second, there is a noteworthy inverse relationship between HDL-C concentration and insulin resistance, which affects SUA levels. Insulin resistance is known to affect UA metabolism, suggesting that changes in HDL-C levels may indirectly affect SUA levels through changes in insulin sensitivity^28^.

In addition, as a combination of PLT and HDL-C, PHR may represent inflammatory and prothrombotic states, both of which are strongly associated with hyperuricemia^20^.

Inflammation significantly influences the onset of hyperuricemia. Research findings have revealed that factors such as the dietary inflammatory index, C-reactive protein (CRP) levels, the Systemic Inflammatory Response Index (SIRI), and the ratio of monocytes to HDL-C (MHR) all have a direct positive relationship with the condition of hyperuricemia^29–32^. During the inflammatory response, platelets engage with neutrophils and lymphocytes, prompting the adhesion and movement of monocytes, which leads to the release of a range of inflammatory mediators. These inflammatory cytokines could potentially enhance the activity of xanthine oxidase through the upregulation of its gene expression, resulting in an increase in UA production^33^. HDL-C has been confirmed as a protective factor against the elevation of serum uric acid in multiple studies, primarily due to its anti-inflammatory effects^18,34,35^. HDL inhibits endothelial inflammation and reduces the expression of key cellular adhesion molecules^36^. Apolipoprotein A-1, a major component of HDL-C, exerts anti-inflammatory effects by decreasing the activation of CD11b on monocytes^37^. UA, in turn, induces inflammation, which is the main driving mechanism for the various complications of hyperuricemia^38^. Even at physiologic concentrations, SUA promotes inflammatory responses and impairs vascular endothelial cell proliferation/migration and nitric oxide (NO) release^39^. Several in vitro experiments confirm this. In rat vascular endothelial cells studied in vitro, UA triggers inflammatory pathways, leading to an increased expression of COX-2 and the chemokine monocyte chemotactic protein-1 (MCP-1)^40^. In human liver cancer HepG2 cells, it was discovered that UA enhances the expression of CRP in a dose-dependent manner.^41^. In addition, because of the free radical-scavenging and antioxidant properties of serum urate, elevated levels of UA may also be a compensatory mechanism designed to counteract the oxidative damage and inflammatory responses associated with atherosclerosis and aging^42^. UA-induced inflammation triggers activation of the coagulation system. Multiple research efforts have documented a connection between SUA levels and the incidence of thrombotic events. One of the first studies to report an association between SUA levels and venous thromboembolism (VTE) was the Atherosclerosis Risk in Communities (ARIC) study, which analyzed 14,126 participants, including 632 cases of VTE events, and found that SUA levels were positively associated with these cases after adjusting for multiple confounders^14^. Other studies have also found that elevated SUA levels are associated with VTE severity, thrombus recurrence, and left atrial thrombosis^13,39,43,44^. Inflammation induces activation of coagulation by damaging the vascular endothelium and upregulating the expression of tissue factor on the surface of circulating monocytes and neutrophils^45^. Elevated UA levels contribute to the upregulation of let-7c, which activates the (myocyte enhancer factor-2c) MEF2C and nuclear factor-κB (NF-κB) pathways, leading to thrombosis^46^. Thus, PHR is strongly associated with hyperuricemia, but it is not clear whether it is a pathogenic risk factor or simply a marker for hyperuricemia.

### Study strengths and limitations

This study demonstrates two primary strengths that enhance the credibility and efficacy of its outcomes. Firstly, the substantial sample size and the consistency between the initial analysis and sensitivity analysis results underscore the robustness of our findings. Secondly, the use of curve fitting has uncovered a nonlinear relationship between PHR and hyperuricemia, suggesting that PHR within an appropriate range (< 8.8) could be advantageous for predicting hyperuricemia.

However, there are limitations to acknowledge. The cross-sectional nature of the study inhibits any deduction of causality. Moreover, due to the observational design, despite adjustments for selected variables, the potential for unaddressed confounding factors exists. Another limitation of this study is the absence of detailed information on participants with blood or liver diseases, which could affect platelet counts. The participants of the study were solely American adults, which may restrict the applicability of the results to other geographical and ethnic groups. Prospective cohort studies are necessitated to corroborate our findings.

## Conclusion

PHR is positively correlated with the risk of hyperuricemia. This discovery underscores the potential of PHR as a biomarker, which could be used to improve the management of hyperuricemia.

## Data Availability

This data can be found here: https://www.cdc.gov/nchs/nhanes.
